# Supplementary material for: Ulvan-Na, an Ulvan Subjected to Na+ Cation Exchange, Improves Intestinal Barrier Function in Age-Related Leaky Gut
Source: Mar Drugs. 2025 Sep 30;23(10):390. doi: 10.3390/md23100390 (PMC12565729; doi:10.3390/md23100390)
Supplement: Supplementary file 1 [file marinedrugs-23-00390-s001.zip › marinedrugs-3869893-supplementary.pdf]

marine drugs

MDPI

Article

# Ulvan-Na, an ulvan subjected to Na⁺ cation exchange, improves intestinal barrier function in age-related leaky gut.

Yuka Maejima [†,1], Yuki Morioka [1], Yusei Sato [1], Masanori Hiraoka [2,3], Ayumu Onda [4] and Takushi Namba [†,1,3,4,5,*]

## Supplementary Materials

**Supplementary Table S1. primer list.**

| | | |
|---|---|---|
| **Primer list for Mouse** | | |
| *Cldn1* | 5'-TCTACGAGGGACTGTGGATG-3' | 5'-TCAGATTCAGCAAGGAGTCG-3' |
| *Cldn2* | 5'-GGCTGTTAGGCACATCCAT-3' | 5'-TGGCACCAACATAGGAACTC-3' |
| *Cldn3* | 5'-AAGCCGAATGGACAAAGAA-3' | 5'-CTGGCAAGTAGCTGCAGTG-3' |
| *Cldn4* | 5'-CGCTACTCTTGCCATTACG-3' | 5'-ACTCAGCACACCATGACTTG-3' |
| *Cldn7* | 5'-AGGGTCTGCTCTGGTCCTT-3' | 5'-GTACGCAGCTTTGCTTTCA-3' |
| *Cldn8* | 5'-GCCGGAATCATCTTCTTCAT-3' | 5'-CATCCACCAGTGGGTTGTAG-3' |
| *Cldn12* | 5'-GTCCTCTCCTTTCTGGCAAC-3' | 5'-ATGTCGATTTCAATGGCAGA-3' |
| *Cldn15* | 5'-CAGCTTCGGTAAATATGCCA-3' | 5'-CAGTGGGACAAGAAATGGTG-3' |
| *Zo-1* | 5'-AGGACACCAAAGCATGTGAG-3' | 5'-GGCATTCCTGCTGGTTACA-3' |
| *Ocln* | 5'-GCTGTGATGTGTGTGAGCTG-3' | 5'-GACGGTCTACCTGGAGGAAC-3' |
| *Jam1* | 5'-ACCCTCCCTCCTTTCCTTAC-3' | 5'-CTAGGACTCTTGCCCAATCC-3' |
| *Muc2* | 5'-AAACTGCTCTCTGGACTGCC-3' | 5'-TTGGTTGGTGTGCTGAGTGT-3' |
| *Lypd8* | 5'-ACCATTTTGCAAGCCAGTGC-3' | 5'-CGCAGAGTGTTTTGTTGTGACC-3' |
| *SOD2* | 5'-GGCTGGETTGGCTTCAATAA-3' | 5'-AATCCCCAGCAGOGOAATAA-3' |
| *IL-1β* | 5'-GATCCCAAGCAATACCCAAA-3' | 5'-GGGGAACTCTGCAGACTCAA-3' |
| *IL-6* | 5'-CTGGAGTCACAGAAGGAGTGG-3' | 5'-GGTTTGCCGAGTAGATCTCAA-3' |
| *Tnf-α* | 5'-CGTCAGCCGATTIGCTATCT-3' | 5'-CGGACTCCGCAAAGTCTAAG-3' |
| *Tlr4* | 5'-CTACCTGGAATGGGAGGACA-3' | 5'-CCAAGTTGCCGTTTCTTGTT-3' |
| *β-actin* | 5'-TTGCTGACAGGATGAGAAG-3' | 5'-ACATCTCTGGAAGGTGGAC-3' |
| **Primer list for Human** | | |
| *CLDN1* | 5'-AAGTGCTTGGAAGACGATGA-3' | 5'-CTTGGTGTTGGGTAAGAGGTT-3' |
| *CLDN2* | 5'-TGCGACACACAGCACAGGCATCAC-3' | 5'-TCAGGAACCAGCGGCGAGTAGA-3' |
| *CLDN3* | 5'-TCATCGGCAGCAGCATCATCA-3' | 5'-ACGATGGTGATCTTGGCCTTG-3' |
| *CLDN4* | 5'-TGCCTGGAGGATGAAAGCG-3' | 5'-GAAGTCTTGGATGATGTTGTGGG-3' |
| *CLDN5* | 5'-CTGGACCACAACATCGTGA-3' | 5'-CACCGAGTCGTACACTTTGC-3' |
| *CLDN7* | 5'-CGGGCGACAACATCATCAC-3' | 5'-GTGGCGACAAACATGGCTAAGA-3' |
| *CLDN12* | 5'-CGGATGAGGCTAGGAGTTTGTCT-3' | 5'-CCAGCGCATGAGCACTACCTG-3' |
| *CLDN15* | 5'-GCCTTGGGATGGTGGCTATCTCG-3' | 5'-TGGTGGCTGGTTCCTCCTT-3' |
| *ZO-1* | 5'-ATCCCTCAAGGAGCCATTC-3' | 5'-CACTTGTTTTGCCAGGTTTTA-3' |
| *OCLN* | 5'-TCCTATAAATCCACGCCGGTTC-3' | 5'-CTCAAAGTTACCACCGCTGCTG-3' |
| *SOD2* | 5'-TGGCCAAGGGAGATGTTACA-3' | 5'-CTTCCAGCAACTCCCCTTTG-3' |
| *IL-1β* | 5'-CTGTCCTGCGTGTTGAAAGA-3' | 5'-TTGGGTAATTTTTGGGATCTACA-3' |
| *IL-6* | 5'-CCAGCTATGAACTCCTTCTC-3' | 5'-GCTTGTTCCTCACATCTCTC-3' |
| *β-actin* | 5'-GGACTTCGAGCAAGAGATGG-3' | 5'-AGCACTGTGTTGGCGTACAG-3' |

 https://doi.org/10.3390/md23100390

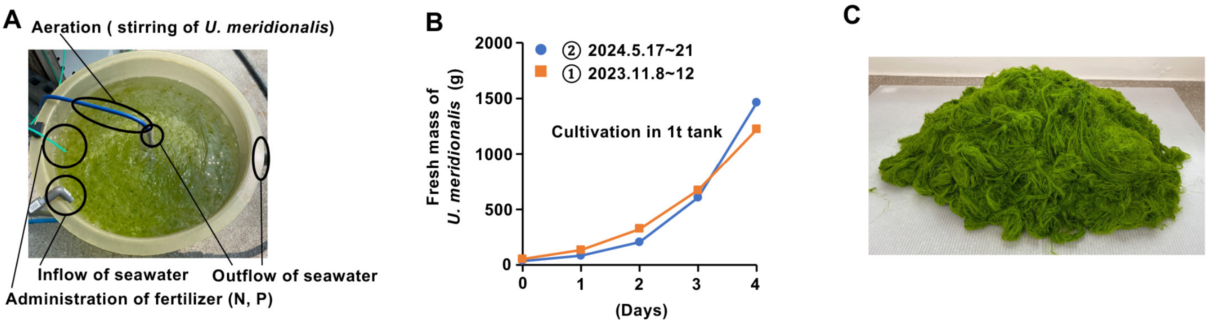


**Figure S1. Outdoor tank aquaculture of *U. meridionalis*.**

(A) Outdoor tank aquaculture system. (B) The fresh mass of *U. meridionalis* was measured daily. (C) Fresh *U. meridionalis*.

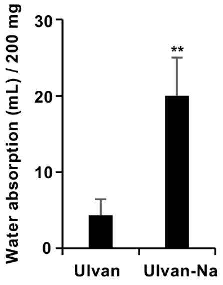


**Figure S2. Compared with ulvan, ulvan-Na increased water adsorption.**

Water adsorption of ulvan and ulvan-Na was determined via a T-bag assay. The data are presented as the means ± SDs of three simultaneous experiments performed in three independent plates. *p* values were calculated via Student's *t* test; **p < 0.01.

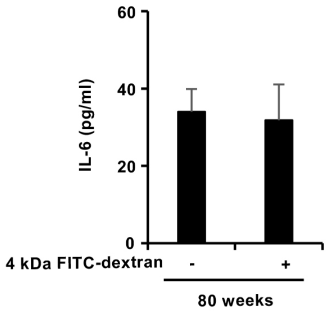


**Figure S3. 4 kDa FITC-dextran did not increase serum IL-6 levels beyond those associated with aging.**

Mice aged 80 weeks were orally administered D.W. (n=5) or 200 mg/kg 4 kDa FITC-dextran for 2hr. Serum IL-6 concentrations were measured.

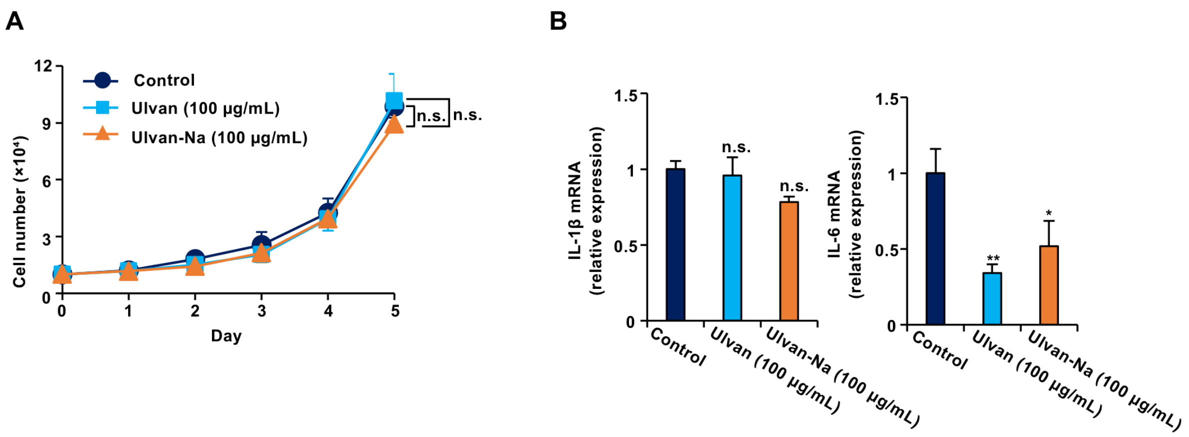


**Figure S4. Ulvan-Na did not affect cellular growth.**

(A) Cell viability was determined via a cell counting assay. (B) Ulvan and Ulvan-Na did not induce IL-1β and IL-6 in Caco-2 cells. The data are presented as the means ± SDs of three simultaneous experiments performed in three separate plates. $p$ values were calculated via Student's $t$ test; n.s.: not significant.
